# Present and future of gait assessment in clinical practice: Towards the application of novel trends and technologies

**DOI:** 10.3389/fmedt.2022.901331

**Published:** 2022-12-16

**Authors:** Abdul Aziz Hulleck, Dhanya Menoth Mohan, Nada Abdallah, Marwan El Rich, Kinda Khalaf

**Affiliations:** ^1^ Mechanical Engineering Department, Khalifa University, Abu Dhabi, United Arab Emirates; ^2^ School of Mechanical and Aerospace Engineering, Monash University, Clayton Campus, Melbourne, Australia; ^3^ Weill Cornell Medicine, New York City, NY, United States; ^4^ Biomedical Engineering Department, Khalifa University, Abu Dhabi, United Arab Emirates; ^5^ Health Engineering Innovation Center, Khalifa University, Abu Dhabi, United Arab Emirates

**Keywords:** clinical gait assessment, gait technologies, gait measures, mobile gait lab, gait pathologies

## Abstract

**Background:**

Despite being available for more than three decades, quantitative gait analysis remains largely associated with research institutions and not well leveraged in clinical settings. This is mostly due to the high cost/cumbersome equipment and complex protocols and data management/analysis associated with traditional gait labs, as well as the diverse training/experience and preference of clinical teams. Observational gait and qualitative scales continue to be predominantly used in clinics despite evidence of less efficacy of quantifying gait.

**Research objective:**

This study provides a scoping review of the status of clinical gait assessment, including shedding light on common gait pathologies, clinical parameters, indices, and scales. We also highlight novel state-of-the-art gait characterization and analysis approaches and the integration of commercially available wearable tools and technology and AI-driven computational platforms.

**Methods:**

A comprehensive literature search was conducted within PubMed, Web of Science, Medline, and ScienceDirect for all articles published until December 2021 using a set of keywords, including normal and pathological gait, gait parameters, gait assessment, gait analysis, wearable systems, inertial measurement units, accelerometer, gyroscope, magnetometer, insole sensors, electromyography sensors. Original articles that met the selection criteria were included.

**Results and significance:**

Clinical gait analysis remains highly observational and is hence subjective and largely influenced by the observer's background and experience. Quantitative Instrumented gait analysis (IGA) has the capability of providing clinicians with accurate and reliable gait data for diagnosis and monitoring but is limited in clinical applicability mainly due to logistics. Rapidly emerging smart wearable technology, multi-modality, and sensor fusion approaches, as well as AI-driven computational platforms are increasingly commanding greater attention in gait assessment. These tools promise a paradigm shift in the quantification of gait in the clinic and beyond. On the other hand, standardization of clinical protocols and ensuring their feasibility to map the complex features of human gait and represent them meaningfully remain critical challenges.

## 1. Introduction

Changes in the signature of gait, or the unique sequential walking pattern in humans, reveal key information about the status and progression of numerous underlying health challenges, from neurological and musculoskeletal conditions to cardiovascular and metabolic disease, and to ageing-associated ambulatory dysfunction and trauma. Accurate reliable identification of gait patterns and characteristics in clinical settings, as well as monitoring and evaluating them over time, enable effective tailored treatment, inform predictive outcome assessment, and an allow for an overall better practice of precision medicine.

In clinical gait assessment, both a person's “ability” to walk and “how” the individual walks are highly relevant. The walking ability of a person is typically based on two main aspects: how far can an individual walk and what is his/her tolerance level (1). For example, for post stroke gait assessment, 3-, 6-, or 10 min walk tests are used, in addition to Functional Ambulation Category (FAC), Short Physical Performance Battery (SPPB), and/or Motor Assessment Scale (MAS). Other clinical subjective assessment scales include the Unified Parkinson Disease Rating Scale (UPDRS) the Scale for the Rating and Assessment of Ataxia (SARA)], the Alzheimer's Disease Assessment Scale (ADAS), the Expanded Disability Status Scale (EDSS) the High-level MobilitARTIy Assessment Tool (HiMAT), and the Dynamic Gait Index (2). The quality of gait or “how” the person walks, on the other hand, highly depends on the quantification of gait patterns and accurate identification of specific gait characteristics. Despite evidence of the advantages of quantitative instrumented gait analysis (IGA) in clinical practice and recommendations by the National Institute for Health and Clinical Excellence (NICE) (3) identifying IGA is the preferrable choice for “gait-improving surgery”, it remains not well leveraged in clinical settings due to the high cost/cumbersome equipment and complex protocols/data analysis associated with traditional gait labs, as well as diverse training, experience and preference of clinical teams (3–5). Moreover, the use of IGA by allied health professionals, such as physiotherapists, occupational therapists and orthotists, and training also remain non standardized and limited (5–7).

Observational gait analysis continues to be popular among clinicians due to its inherent simplicity, availability, and low cost (8). On the other hand, the validity, reliability, specificity, and responsiveness (9, 10) of these qualitative methods are controversial and increasingly being questioned (6). Furthermore, there is evidence to suggest that subjective clinical assessment scales may not be sensitive to disease severity and specific characteristics and may limit understanding of underlying disease mechanisms, hence adversely impacting optimal treatment (11). Examples of such scales include Multiple Sclerosis (MS), where subjective measures, such as the Expanded Disability Status Scale (EDSS), the Multiple Sclerosis Severity Scale (MSSS), Multiple Sclerosis Walking Scale (MSWS), and Multiple Sclerosis Functional Composite (MSFC), continue to be widely used in clinical practice. These scales have been criticized for lack of sensitivity (12), high interrater variability (13), as well as being prone to practice effects and variability (14, 15). Similarly, clinical assessment of Parkinson's disease (PD) using the Unified Parkinson's Disease Rating Scale (UPDRS) is subjective and largely dependent on the expertise and experience of the clinicians, as well as the severity of the disease (16). In Stroke patients, assessment tests such as Functional Ambulation Category (FAC), Short Physical Performance Battery (SPPB), and/or Motor Assessment Scale (MAS) are typically employed, along with qualitative observational/visual gait analysis (using naked eye or video images). Nevertheless, the validity, reliability, specificity, and responsiveness of these qualitative methods are questioned (9), and although they may be useful for the rudimentary evaluation of some gait parameters, they are not adequate for analyzing the multifaceted aspects of gait variability and complexity (17).

Instrumented gait analysis (IGA), which can provide accurate and precise quantitative measurement of gait patterns and characteristics, has long been the gold standard for gait assessment in research practice (18). IGA generally refers to the use of instrumentation to capture and analyze a variety of human gait parameters (spatiotemporal, kinematic, and kinetic measures). Traditional IGA systems include motion capture systems, and force plates, instrumented walkways, and treadmills, while more recent systems comprise of miniaturized wearable sensing system, computational platforms and modalities (18). Literature on the clinical applicability and efficacy of IGA indicates that IGA-based quantitative assessment can improve the diagnosis, outcome prediction, and rehabilitation of various gait impairments as compared to conventional observational scales and techniques for gait dysfunction in a wide spectrum of diseases including MS, PD, Stroke, and Cerebral Palsy (9–13). A recent review on the clinical efficacy of IGA confirms that there is strong evidence that 3-D gait analysis, or 3DGA; has the potential to alter and reinforce treatment decisions; increases confidence in treatment planning and agreement among clinicians; can better identify diagnostic groups and expected treatment outcomes; and overall can improve patient outcomes if recommendations are followed (19).

Emerging at an unprecedented rate, wearable sensing systems and associated computational modalities are rapidly transforming the quality and accessibility of healthcare, spanning multiple applications from neurology and orthopedics to cardiovascular, metabolic, and mental health. Magnetic (e.g., magnetometers), inertial measurement (e.g., accelerometers and gyroscopes), and force sensors (e.g., insole foot pressure) nowadays offer unprecedented data capture opportunities that can overcome limitations of non-wearable devices due to their low-cost, less setup-time and complexity, lightweight, and portability, making them ideal for out-of-lab and continuous monitoring in the clinic and beyond (20). Magneto-inertial measurement units (MIMUs), in conjunction with force pressure sensors, have the capability of capturing spatiotemporal, kinematic, and kinetic gait data (2) rendering the concept of a mobile gait lab a reality. Such labs can inherently overcome the limitations of IGA traditional labs, providing less costly and cumbersome tools with potential for gait assessment in natural environments (clinics, homes, sports arenas, etc.), user friendly interfaces, and the opportunity to provide continuous real-time feedback to clinicians and patients, as well as tele rehabilitation capabilities. In addition, wearable systems allow for easy synchronization with other physiological measurement systems, including EMG, ECG, and EEG, towards the acquisition of invaluable multimodal continuous physiological data in various settings.

This scoping review aims to provide a summary of the current state of clinical gait assessment, including shedding light on gait pathologies and clinical indices and scales, as well as a roadmap for the development of future gait mobile labs- highlighting the clinical validity and reliability of the latest devices and data interpretation algorithms. The word novel in the title of this review reflects recent emergence/implementation of the technologies reviewed and/or recent commercialization. This includes wearable technologies, as well as AI-driven computational platforms. The remainder of the review is structured as follows: Section 2 describes the adopted methodology, including the approach, search strategy and selection criteria. Section 3 details clinical gait pathologies, relevant parameters, as well as current clinical gait assessment tools, scales, and indices, while Section 4 presents gait assessment technologies applicable to clinical settings, including state-of-the-art imaging techniques and wearable technologies, algorithms, and novel AI-driven computational platforms. Section 5 deliberates on the concept of a mobile gait lab for clinical applications. Section 6 highlights the limitations, while Section 7 presents the conclusive remarks and future work.

## 2. Methods

This review is aimed at summarizing various clinical gait pathologies and associated parameters, applicable gait analysis techniques and gait indices, and the latest trends in wearables systems and algorithms. To address this broader research objective, the authors adopted a scoping review approach rather than a systematic review approach. As reported in (21), scoping reviews are ideal for addressing a broader scope with a more expansive inclusion criterion.

### 2.1. Search criteria

A keyword search was performed in PubMed, Web of Science, Medline, and ScienceDirect databases, using a combination of search terms from the following groups: 1. (normal gait OR pathological gait OR gait parameters OR gait indices), 2. (gait assessment OR gait analysis), 3. (wearable systems OR wearable algorithms), 4. (inertial measurement units OR accelerometer OR gyroscope OR magnetometer OR insole sensors OR electromyography sensors). No limit for the year of publication was set, however, the search was last updated in December 2021. Only articles written in the English language were considered in this review. In addition, the reference list of the included articles was checked to identify additional relevant publications meeting the inclusion criteria. The literature search and data extraction were carried out independently by two authors (AAH, DMM) and any inconsistencies and disagreements discrepancies were resolved through following discussions with the other authors (NA, MER, KK).

**Figure 1:**
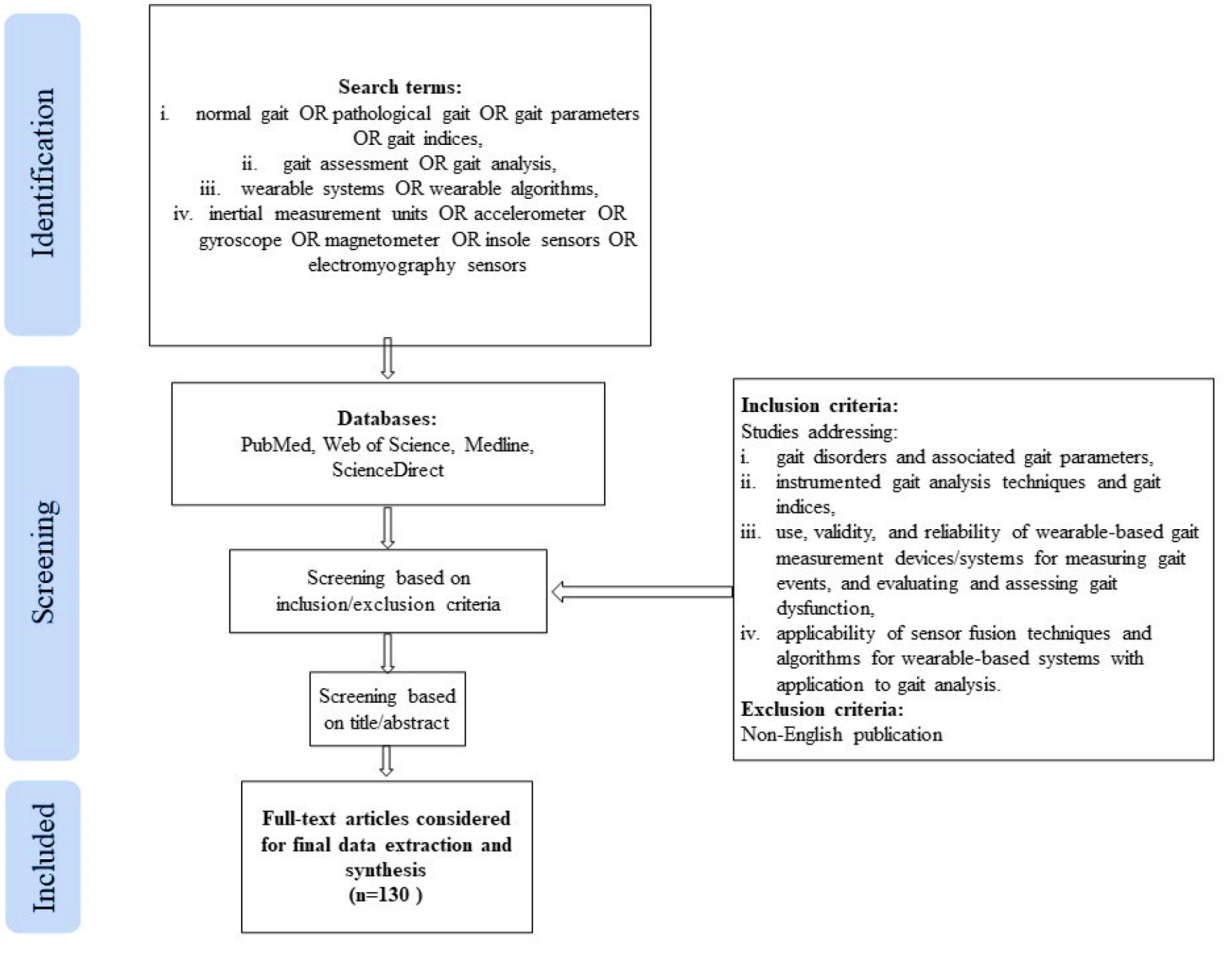
Publication selection process.

This scoping review included original published works and review articles which met the following inclusion/exclusion criteria: (i) studies addressing various gait disorders and associated gait parameters, (ii) studies focusing on instrumented gait analysis techniques and gait indices, (iii) studies evaluating the use, validity, and reliability of wearable-based gait measurement devices/systems for measuring gait events, and evaluating and assessing gait dysfunction, (iv) studies concerning the applicability of sensor fusion techniques and algorithms applicable for wearable-based systems with application to gait analysis. The title and/or abstract of the studies were initially screened for suitability. The full-text articles meeting the inclusion criteria were obtained for data extraction and synthesis. A flowchart explaining the same is shown in Figure 1.

## 3. Clinical gait pathologies and parameters

### 3.1. Normal gait cycle and parameters

**Figure 2:**
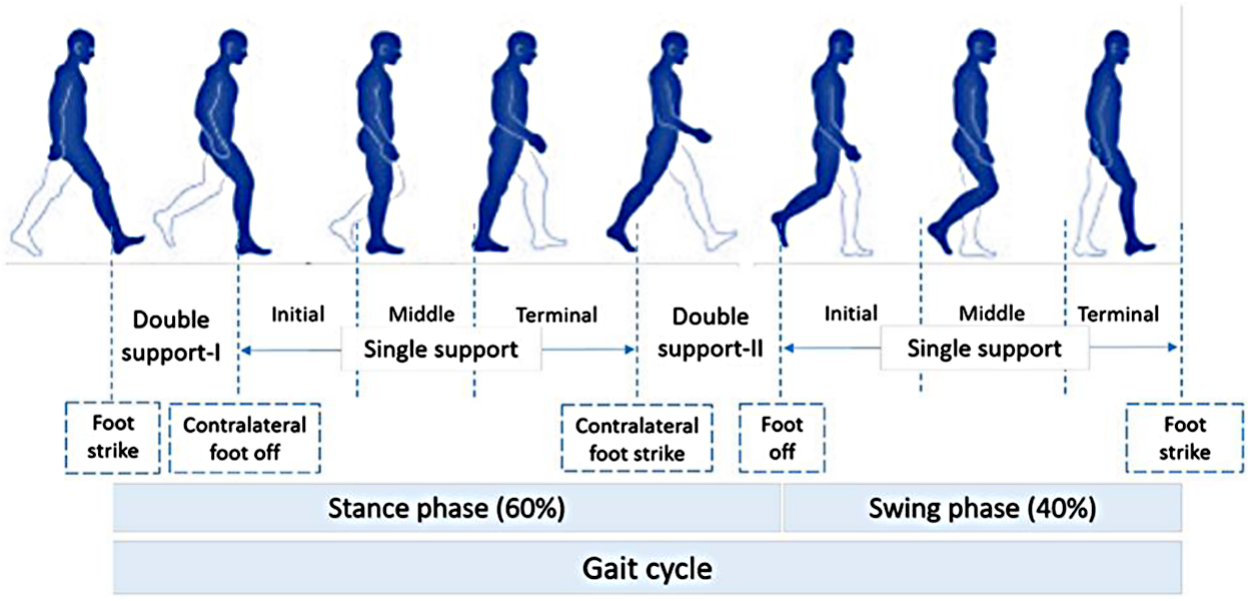
Normal gait cycle (adapted from 1).

Normal gait can be defined as a series of rhythmic, systematic, and coordinated movements of the limbs and trunk that results in the forward advancement of the body's center of mass (22). A result of intricate dynamic interactions between the central nervous system and feedback mechanisms (23), walking is characterized by individual gait cycles and functional phases (Figure 2). A gait cycle consists of two main phases, stance, and swing, which are further divided into five and three functional phases, respectively. The stance phase corresponds to the duration between heel strike and toe-off of the same foot, constituting approximately 60% of the gait cycle. The swing phase begins with toe-off and ends with heel contact of the same foot and occupies 40% of the cycle. As each functional phase contributes to successfully accomplishing the goal of walking, healthy gait involves cyclic and complementary movements of the limbs under control. It is characterized by stance stability; toe clearance during the swing; pre-positioning at swing; sufficient step length; as well as mechanical and metabolic efficiency (24). Table 1 provides gait parameter ranges based on studies on healthy adults. Determining an appropriate normal range for many of the features is highly challenging as individuals exhibit a wide range of gait patterns across different age groups and gender (17).

**Table 1:** Gait parameters for healthy individuals (1).

Parameters (self-selected speed)	Range
Gait velocity (m/s)	1.30–1.46
Stride length (m)	1.68–1.72
Step length (m)	0.68–0.85
Stance phase (s)	0.62–0.70
Swing phase (s)	0.36–0.40
Cadence – fast walking (steps/min)	113–118
Single support (% of stride)	60.6–62
Double Support (% of stride)	21.2–23.8

### 3.2. Gait parameters associated with pathology

Gait disorders are typically associated with deficits in the brain, spinal cord, peripheral nerves, muscles, joints, or bones. Some medical conditions leading to pathological gait include but not limited to muscular dystrophy, myelodysplasia, cerebral palsy, arthritis, osteoarthritis, head injury, lower limb amputation, multiple sclerosis, rheumatoid, spinal cord injury, parkinsonism, and stroke (25).

In neuromuscular conditions, the loss of central control affects the motion. In general, patients walk slower than healthy individuals and with compromised spatiotemporal, kinematic, and kinetic parameters. In older adults, a walking speed decline of 0.7% per year is observed, along with significant changes in cadence and step length. The aging population also exhibits lower knee extension at heel-strike and knee flexion during the swing phase (23, 26). The following subsections describe some of the most common gait disorders and associated pathological parameters. The associated impacted parameters are summarized in Table 2.

**Table 2:** Effect of pathology on gait disorders.

Pathology	Impacted Gait Parameters
1. Neurological gait disorders in elderly people	
1.1. Hypokinetic-rigid gait	• Shuffling • Reduced step height • Reduced stride length • Variability in steps
1.2. Cautious gait	• Reduced speed • Wider base • Shorter stride • Knees and elbow bent
1.3. Careless gait	• Walks insensitively fast
1.4. Dyskinetic gait	• Walk on toes
1.5. Psychogenic gait	• Ataxia
2. Parkinson's Disease	• Deficit in amplitude and gait speed • Increased gait variability
3. Diabetic Peripheral Neuropathy	• Extra steps • Reduction in speed, step length, cadence • Reduced joint range of motion at ankle and knee
4. Post Stroke (hemiplegia) gait	• Asymmetrical Deficit • Decreased walking speed and cadence • Longer gait cycle and double limb support • Reduced peak extension at hip
5. Hip Osteoarthritis	• Reduced hip range of motion • Reduced velocity
6. Knee Osteoarthritis (122)	• Reduced gait speed • Reduced stride length • Increased step width • Reduced range of motion at Hip, knee, and ankle • Reduced moments in sagittal plane at knee, hip, and ankle • Increased coronal plane moment at knee and ankle, and reduction at hip.

#### 3.2.1. Neurological gait disorders in elderly people

Gait ailments associated with aging lead to reduction in the quality of life and increased morbidity and mortality. Elderly patients exhibit complex gait disorders, and their dual task ability deteriorates due to a decline in their central resources (23, 26).

Specific gait dysfunction noted in the elderly population are summarized as follows:

##### 3.2.1.1. Hypokinetic-rigid gait disorders

Shuffling with a reduced step height and stride length characterizes hypokinetic gait disorder (27). Reduced arm swing with slow turning movements is also present in isolation. Festination, when patients use rapid small steps to maintain the feet beneath the forward moving trunk, is also observed. Ataxic elements include broad stance width and an increased variability in timing and amplitude of steps (27). Gait associated with underlying diseases, such as Parkinson's disease, cerebrovascular disease, and ventricular widening, is classified within hypokinetic-rigid gait disorders (27, 28).

##### 3.2.1.2. Cautious and careless gait

Defined as gait during which people move slowly with a wider base, and shorter stride, with minimal trunk movement, while the knees and elbows are bent. Whereas careless gait is when patients appear overly confident and walk insensitively fast. Careless gait is due to confusion and delirium associated with old age (27).

##### 3.2.1.3. Dyskinetic gait or involuntary movements

Patients with post-anoxic encephalopathy exhibit bouncing gait and stance. This is also observed in patients with Parkinson's disease-causing excessive trunk movements contributing to falls. Several dystonic patients are reported to walk on their toes (27, 28).

##### 3.21.1.4. Psychogenic gait disorders

Gait dysfunction is common in elderly people due to adverse effects of drugs leading to extrapyramidal side-effects, sedation, orthostatic hypotension, behavioral abnormalities, or ataxia (27, 28).

##### 3.2.1.5. Fluctuating or episodic gait disorders

Elderly people often exhibit fluctuating or episodic gait disorder after exercise due to fatigue, and it might be an indication of underlying vascular or neurogenic limping. Freezing gait is part of hypokinetic-rigid syndrome (27, 28).

#### 3.2.2. Gait disorders in Parkinson's disease

PD is a neurological disorder which leads to cognition, where gait impairment deteriorates with disease progression, increasing reliance on cognition to control gait. Due to cognitive impairment with PD, the ability to compensate for gait disorders diminishes, leading to further gait impairment. PD is characterized by deficit in amplitude and gait speed, along with increased gait variability (29).

#### 3.2.3. Gait in diabetic peripheral neuropathy

Neuropathy of motor, sensory, and autonomic components of the nervous system are one of the many complications of Type II Diabetes (T2D). An intact central and peripheral nervous system are essential to initiate and control healthy gait, along with sufficient muscle strength, bone, and joint movements in complete range for normal locomotion. Patients diagnosed with T2D take extra steps when walking in straight paths and during turns, along with an overall reduction in walking speed, step length, cadence, and fewer acceleration patterns as compared to age-matched healthy controls. Joint range of motion is also altered in T2D, where patients with diabetic peripheral neuropathy exhibit a reduced range of motion at the ankle joint in dorsi and plantar flexion and a reduced flexion and extension range of motion at the knee joint in both, as compared to non-diabetic people (24).

#### 3.2.4. Post stroke gait

Hemiplegia after stroke contributes to significant reduction in gait performance. In stroke survivors, function of the cerebral cortex is usually impaired, whilst that of spinal cord is preserved. Dysfunction is typically demonstrated by a marked asymmetrical deficit. Decreased walking speed and cadence, in addition to longer gait cycle and double limb support as compared to healthy individuals. For hemiplegic stroke survivors, a reduced peak extension of the hip joint in late stance, varying peak lateral pelvis displacement, knee flexion and decreased plantarflexion of ankle at toe off are reported. The GRF (Ground Reaction Force) pattern is characterized as asymmetric, along with decreased amplitude of joint moments, at the lower limb joints on the paretic side (30).

#### 3.2.5. Total hip arthroplasty (THA)

Large deficits in gait speed (31), stride length (32, 33), sagittal hip range of motion (32, 33), hip abduction moment-coronal plane (31), and negligible changes in transverse plane hip range of motion (31), deficiency in single limb support time (31), are reported in patients post THA as compared to healthy controls. Peak hip extension is typically reduced, whereas peak hip flexion remains similar as compared to controls. In addition, peak hip abduction moment is reduced along with peak hip external rotation moment (34).

### 3.3. Clinical gait assessment measures and indices

The use of observational gait analysis and subjective rating sales continues to be widespread in clinical settings, both as a diagnostic tool and as a prognostic measure, as previously mentioned. Although these techniques can be useful for the initial rudimentary evaluation of some gait parameters, the validity, reliability, specificity, and responsiveness of these qualitative methods are highly questionable. Researchers have therefore proposed various pathology-specific gait indices and summary measures (35) based on commercially available technologies with accepted levels of accuracy Table 3. summarizes the current clinical gait summary measures, discrete and continuous gait indices, and non-linear approaches reported in literature, along with advantages and disadvantages.

**Table 3:** Clinical gait measures and indices (123, 124).

Gait measures/indices	Normal range	Statistical method	Parameters			Index provides	Advantages	Disadvantages
			Spatio-temporal	Kinematic	Kinetic			
Normalcy Index/Gillette Gait Index (GGI)	Euclidean distance between pathological and normal gait -Smaller the value closer to baseline	Principal Component Analysis (PCA)	1. Percentage of stance phase, 2. Normalized velocity 3. Cadence	1. Mean pelvic tilt, 2. range of pelvic tilt, 3. mean pelvic rotation, 4. minimum hip flexion, 5. range of hip flexion, 6. peak abduction in swing, 7. mean hip rotation in stance 8. knee flexion at initial contact, 9. time of peak knee flexion, 10. range of knee flexion, 11. peak of dorsiflexion, 12. peak of dorsiflexion in swing, 13. mean foot progression angle	-	Indicates amount by which a subject's gait deviates from an average normal profile	1. Extensively validated, 2. Quantify effect of treatments that have a global effect on gait pattern.	1. Lack of appropriate specificity and sensitivity when evaluating effects of targeted interventions, 2. Arbitrary, unbalanced, and incomplete nature of 16 univariate parameters, 3. Absence of Kinetic variables, 4. Only characteristic points of the curves are included, 5. Strongly sensitive of lab-specific control data, 6. Minimum sample size required in set of control subjects to have reliable NI tool.
Hip Flexor Index	–	Principal Component Analysis (PCA)	–	1. Maximum pelvic tilt, 2. pelvic tilt range, 3. maximum hip extension in stance,	1. Percentage of stance phase in which final crossover of hip flexor moment curve from extension to flexion, 2. peak late stance hip flexor power	Describes overall hip function during gait	1. Valid tool to objectify clinical impressions of a change in hip function, 2. Measures post-operative change in hip function corresponding with subjective clinical outcome 75%	1. Joint Specific, 2. May or may not signify global improvement in patient's gait, 3. No correlation between change in score on HFI and change in function of patient
Gait Deviation Index (GDI)	Normal GDI >100 Abnormal GDI < 100 (Every 10 points less than 100 is 1 Standard deviation from baseline)	Singular Value Decomposition	–	1. Pelvis and hip kinematics in 3 planes, 2. Knee and ankle on sagittal plane 3. Foot progression	–	Measure of extent of gait pathology from control group.	1. Moderately correlated to NI, 2. Demonstrated distributional properties for adults as those reported in studies on healthy and ambulant children with cerebral palsy, 3. Entire variability in kinematic variables across gait cycle is used, 4. More general measures of gait pathology are taken, 5. Values of GDI are less sensitive to differences in reference data	1. GDI fails to identify significant differences between the levels of functional limitations of the intact side for limb amputees, 2. GDI was reportedly not sufficiently sensitive to measure gait severity in Parkinson's patients without levodopa medication, 3. No spatio-temporal parameters and kinetics were included
Gait Profile Score and movement analysis profile	–	Feature Analysis, Bar charts, Gait variable score (GVS) and Movement Analysis Profile (MAP)	–	1. Pelvis and hip kinematics in three planes 2. Knee and ankle on sagittal plane, Foot Progression	–	Difference between patient's data and average from reference dataset	1. Validated against established index measures of gait abnormality and general measures of mobility in children with CP, 2. Provides MAP and GVS, 3. Compares against a single column of able-bodied data	1. Fewer reports of clinical use, 2. No spatial temporal and kinetics parameters were included
GDI-Kinetic	Normal GDI >100 Abnormal GDI < 100 (Every 10 points less than 100 is 1 Standard deviation from baseline)	Singular Value Decomposition	–	–	20 gait features of raw gait kinetic data	Measure of gait pathology from control group based on joint kinetics	1. Assess the mechanisms that either control or produce the movement, 2. Comprehensive understanding of motion, 3. Complements GDI	1. No studies and applications in literature
**Discrete Symmetry Index**								
Ratio Index (symmetry ratio)	= 1 perfect theoretical symmetry < or > 1, asymmetry	Ratio of gait parameter values for one limb over the contralateral limb	Gait features like step length, single support phase duration, swing duration, stride time, cadence, stride height.	–	–	Measure of asymmetry between the 2 limbs, value 1 represents symmetric gait	simple to use	1. No upper limit, 2. Difference in symmetry results based on numerator or denominator, 3. Discrete time events, do not describe complete wave forms
Robinson Index (symmetry index)	= 0. Symmetric gait ≠ 0, asymmetric gait		Gait phases duration, step length, cadence, COP maximum displacement, step width, single limb support time, arm swing duration, swing phase duration	Length of forward foot placement relative to trunk and trunk progression of paretic and nonparetic steps, range of arm swing angle, lower limb angular rate.	Ground reaction force, muscle activity, and power value	Value 0 indicates symmetrical gait, divergence from zero means asymmetry	–	1. Unlimited lower and upper bounds of index, 2. variable specific and not a valid criterion to assess gait asymmetry when using several gait variables, 3. Discrete time events, do not describe complete wave forms,
Symmetry Angle	–		Stride duration	Standard deviation of arm angular acceleration, wrist displacement, max trunk angular rotation	Peak GRF	–	Standard scale for interpretation of calculated asymmetry	Discrete time events, do not describe complete wave forms
**Continous Symmetry Index**								
Trend Symmetry	0, perfect symmetry Range 0-1	Eigenvectors to compare time series	–	Joint angles	Force moment profiles	Ranges from 0 to 1, 0 indicates perfect symmetry	Effectively identifies joint angle behaviors i.e., no longer symmetric	Does not identify timing in gait cycle when these deviations are occurring
Geometric properties of cyclograms	Ideal point I (0.45,0)	Cyclograms	–	–	–	Ideal point I, symmetric gait has (0,45,0) coordinates, Asymmetry is defined as Euclidean distance from measurement point M and ideal point I.	–	There is no upper limit, higher the symmetry value higher the level of asymmetry
Region of deviations	–	Difference between affected and unaffected sides of a given joint computed over hait cycles	–	Joint angles, Range of motion	–	Average difference compared to joint motion from normative subjects	Possible to identify which joints are affected during gait, the timing and magnitude of joint angles during gait cycle, when combined with another method	Not conclusively indicate which joint is most affected, Need for normative data from able-bodied subjects as reference
Symbol-based method	–	Symbolic representation of time series, comparison between histograms of right and left signals	–	–	Symmetry value ranges from 0 to 100, 0 indicates perfect symmetry	Used as a base to gait event detection	Method input parameters influence the results and need to be determined prior	–
Non-linear approaches								
Multiresolution entropy	1, perfect symmetry 0, asymmetry	Wavelet transformation to decompose original data into multi levels and modified sample entropy is calculated at each level from a pair of time series	Stance time	–	–	Symmetry value ranges from 0 to 1, 1 indicates perfect symmetry	–	Parameters need to be set up prior to method utilization, Parameter values might be critical for method outcomes
Cross-fuzzy entropy	–	Derived from fuzzy entropy based on concept of fuzzy sets	Stride interval	–	–	–	–	Three parameters need to be set up prior to method utilization

## 4. Gait assessment technologies applicable to clinical settings

In the past couple of decades, remarkable technological advancement has been witnessed in the field of gait assessment and analysis, particularly in gait assessment technology. Instrumented walkways, both portable and non-portable, became a good alternative to complicated, bulky and non-portable traditional gait labs. These systems (for example the Walkway and StrideWay from Tekscan Inc., Boston, United States) are now widely used in research and to a limited extent in clinical practice. They typically include low-profile floor walkway systems equipped with grids of embedded sensors below the surface, which record foot-strike patterns as a function of time and space as an individual walks across the platform, and dedicated software which computes the various spatiotemporal gait measures Although these instrumented mats involve less setup time and are generally simple to operate as compared to traditional IGA labs, they are expensive, restrictive to specific operational environment to over-ground trials (36, 37).

Marker-based optical motion capture (Mocap) is another rapidly emerging technology effective for obtaining 3D kinematic movement data. Passive Mocap systems [e.g., Vicon (Vicon Motion Systems Ltd, Oxford, United Kingdom) and ELITE optoelectronic system (BTS S.*p*.A., Milano, Italy)], include retro-reflective markers (that reflect the light emitted by high-resolution infrared cameras) attached to specific anatomic landmarks. The location of the marker is identified by decoding the camera images. Here, the markers must be calibrated for identification before the recording session commences. Active Mocap systems (e.g., Optotrak motion capture system; Northern Digital Inc., Waterloo, Canada), on the other hand, use light-emitting diode (LED) markers (reflect their own light powered by a battery), which are automatically identified (38, 39). In the context of clinical relevance, although such systems yield extremely accurate reliable data, operational factors including infrastructure, non-portability, high cost, additional time required for initial set-up and calibration, operational complexity, and restrictions to indoor setup impose hurdles to their functional deployment in clinics and rehabilitation centers (84). Recently, more portable cost-effective alternatives, such as Microsoft Kinect (based on a depth sensor-based markerless motion capture solution) became the application of choice (40).

Optoelectronic systems (e.g., Optogait®, Microgate, Italy) have also been used to capture spatiotemporal gait parameters. These mainly consist of a transmitting and a receiving bar containing an infrared light. Interruptions of the communication between the emitter and receiver are detected by the system to calculate the various gait parameters (41).

An evolution in the measurement of gait kinetic parameters can also be witnessed in the last two decades. These parameters include ground reaction forces, and intersegmental joint reaction forces, moments, and powers. Instrumented walkways offer dynamic plantar pressure mapping but are expensive and do not provide joint kinetic data. Force plates are also used in various gait analysis studies (38, 39, 42). These are able to provide intersegmental joint reaction forces by using the ground reaction forces measured along with inverse dynamics models (Winters book) Chen et al. (93) developed a novel remote sensing technology called “Electrostatic Field Sensing (EFS)” for measuring human gait including stepping, walking, and running, and further extended the work to post-stroke gait. This technology is credited with several advantages, such as being non-contact, affordable, and allows long-time monitoring (43). Shoe insole systems represent another category of gait quantification tools and techniques. These systems are designed to allow for the recording of both dynamic plantar pressure and spatiotemporal data. F-scan (Tekscan Inc., Boston, United States) is an ultra-thin in-shoe pressure measurement system utilizing Force-Sensitive Resistive films (FSR) technology (44).

The characteristics of different measurement systems applicable to clinical settings are summarized in Table 4, and the pros and cons of these systems are listed in Table 5.

**Table 4:** Portable wearable gait assessment tools.

Wearable technology	Gait parameters assessed	Advantages	Disadvantages
Tri-axial Accelerometers	• Assessing stability • Risk of Fall	• Easy implementation in clinical Practice	• Discrepancies in sensors positioning yield
Gyroscopes (used in combination with MEMS devices)	• Step detection • Gait event detection • Segmental Kinematics	• Easy implementation in clinical and home environment	• Could not achieve required positioning accuracy • Large drift Bias
Inertial Measuring Units	• Detect risk of fall • Gait events measurements • Gait symmetry estimation	• Low-cost compared to optical • No restriction to space as in optical	• Distortion of kinematic data caused by drift effect
Shoe Insoles	• Center of pressure trajectories • Plantar pressure monitoring	• Cost Effective • Portable	• Distortions of flexible contact surface due to repeated loading • Drift due to prolonged load application • Need for subject-specific calibration
Electromyography Sensors	• Measure surface muscle activity • Muscle contraction abnormalities	• Useful in CP, stroke disorder diagnosis • Distinguish specific gait abnormalities	• Lack of normative activation patterns

**Table 5:** Pros and cons of different IGA systems.

Instrument	Pros	Cons	Current manufacturers
Pressure mat	Less setup time, easy to operate	High cost, non-portable, restricted to over-ground trials, require specific operational space	Tekscan Inc. (Walkway, F-Mat), Novel Electronics Inc. (EMED)
Pressure insole	Portable, cost-effective, does not require specific operational space, useful for indoor and outdoor setup	Low accuracy compared to pressure mat	Tekscan Inc (F-Scan), Novel Electronics Inc. (Pedar)
Motion capture	Highly accurate, useful for complex tasks involving motion in multiple planes	High cost, non-portable, additional time requirements for initial setup and calibration, special training required for operating the system, restrictions to indoor setup	Northern Digital Inc. (Optotrak), Qualisys (Arqus, Miqus), Vicon Motion Systems Ltd (VCON), BTS S.*p*.A. (Elite, SMART-DX)
Wearable sensors	Low cost, does not require specific operational space, useful for indoor and outdoor setup, less setup and calibration time	Special algorithms required to combine multiple sensor data	Xsens (MTW), Shimmer Sensing (Shimmer3 IMU), GaitUp SA (Physilog)

Computational pipeline using computer vision techniques has been proposed as an ecological and precise method to quantify gait in children with neurodevelopmental disorders, along with the pose estimation software to obtain whole-body gait synchrony and balance (45). Speed, arm swing, postural control, and smoothness (or roughness) of movement features of gait for Parkinson's patients were extracted using videos processed by ordinal random forest classification model. Significant correlation between clinician labels and model estimates was reported, which provides gait impairment severity assessment in Parkinson's disease using single patient video, thereby reducing the need for sophisticated gait equipment (46). Computer vision-based gait assessment tools promise frequent gait monitoring using minimal resources (46). Deep learning to detect human subject in 2D images and then combining 3D sensing data to measure gait features has proven to be more robust than depth cameras in gait parameter acquisition (47).

### 4.1. Imaging techniques for gait assessment

As previously mentioned, marker-based optoelectronic systems are currently the most widely used systems in IGA among both research and clinical communities. On the other hand, one of the main sources of error inherent to these systems is the degree of movement of the skin, muscle, and other soft tissues, or the so- called soft tissue artifacts (STA), under the markers in relation to bony landmarks, hence violating the rigid body assumption underlying these methods (48, 49). Moreover, STA varies by marker location in a unique and unpredictable manner, particularly during dynamic activities, which can make it unreliable for clinical applications (50).

Although not yet widespread in biomechanics, computer vision based markerless gait assessment methods offer a promising tool for gait assessment in research, as well clinical and sports biomechanics applications. By leveraging modern technologies, such as improved solvers, advanced image features and modern machine learning, markerless vision-based systems can reduce the required number of cameras, incorporating moving cameras, increasing the number of tracked individuals, and offering robust detection and fitting in diverse environments. On the other hand, issues such as accuracy and field-based feasibility remain to be addressed (51).

Three-dimensional imaging techniques have been successfully used to directly determine bone movements during walking as a gold reference standard to validate/improve current motion capture techniques (54). For example, researchers have resorted to quantifying STA by comparing with reference 3D kinematics of bone reconstructed from fluoroscopy-based tracking (53). Fluoroscopy has also emerged as a means for tracking position and orientation of underlying skeletal anatomy of the foot/ankle (54). Although single plane fluoroscopy yielded large errors when used to evaluate the accuracy of multi-segment foot models (49), dual fluoroscopy (DF) was found reliable and is considered as the current reference standard to compare joint angles (55). Combined with 2D/3D registration, video-fluoroscopy allows for accurate quantification of 3D joint motion free of STA (56). High-speed dual fluoroscopy (DF) has been reported to measure *in-vivo* bone motion of the foot and ankle with sub-millimeter and sub-degree errors (57). DF has also been used to evaluate multi-segment foot models and reported good agreement between DF and skin-marker data for the first metatarsal and sagittal plane measurements of the longitudinal arch (48).

Various researchers investigated the use of DF for clinical applications*. In-vivo* dual fluoroscopy was used to quantify the hip joint kinematics of patients with Femoroactabular impingement syndrome (FAIS) relative to asymptomatic, morphologically normal control participants during standing, level walking, incline walking and an unweighted functional activity. The kinematic position of the hip joint was obtained by registering projections of 3D computed Tomography models with DF images (58). Knee kinematic profiles were also obtained using 3D video-fluoroscopy and compared to actual and nominal flexion-extension, internal-external rotations, and antero-posterior translations profiles with optical mocap during stair climbing (59). Joint function for total talonavicular replacement after a complex articular fracture was evaluated using a full body gait analysis and 3D joint kinematics based on single-plane fluoroscopy (60). The 3D video fluoroscopic analysis was performed to assess joint motion of the replaced ankle (60). DF and CT imaging techniques were both employed to calculate *in-vivo* hip kinematics, along with model-based tracking, to compare the effect of different coordinate systems (61). Since marker-based systems are unable to accurately analyze talocrural or subtalar motion because the talus lacks palpable landmarks to place external markers (54), digitized video fluoroscopy was reportedly used to determine the sagittal plane motion of the medial longitudinal arch during dynamic gait (62). Characteristics of knee joint motion were also analyzed in 6DOF during treadmill walking using a dual fluoroscopy imaging system at different speeds (63).

DF uses anatomical landmarks visible on 3D CT reconstructions which substantially reduces errors due to STA (58). Computed tomography (CT) scans of participants are usually needed in DF to determine bone position from the DF images. Single plane fluoroscopy is restricted to 2D motion capture, while using a second FS allows for a full 3D analysis although a single gantry system has lower radiation than the biplane system with reported ionizing radiation levels of 10 µSv per trial (54). Stationary image intensifiers and static systems have a restricted field of view limiting their application to highly restricted movements (56). Moving fluoroscopes, consisting of a fluoroscopic unit mounted on a moving trolley which moves with the subject and is controlled by wire sensors to ensure that it remains in the field of view of the image intensifier (56), provide an enhanced field of view ideal for dynamic scenarios and moving joints.

Fluoroscopic systems designed for precise capture of bone movement and joint kinematics, unlike optical or inertial systems, are not yet commercially available, generally requiring in-house instrumentation and further performance evaluation. The evaluation would typically include determining the resolution of the hardware imaging chain, assessing how the hardware and software reduce or eliminate various distortions, and measuring static and dynamic accuracies and precisions based on precisely known motions and positions (64). Image quality is a major determinant of error in fluoroscopic applications (62). Pulse imaging of fluoroscopes, such as pulse width, limits image quality at a given frame rate. Increasing the pulse rate, which is function of pulse width, may add to radiation exposure, leading to an important tradeoff consideration between image quality and radiation exposure (63). Moving video-fluoroscopes reported lower gait velocity, step length, and cadence as compared to control conditions, indicating altered time distance parameters towards those of slow walking (56). So far, dynamic MRI used to define in-*vivo* talocrural and subtalar kinematics (65) does not allow data collection during normal gait.

Continued multidisciplinary collaborative efforts among biomechanists, imaging and computer vision experts, and clinicians are essential for fully leveraging these highly promising techniques in clinical applications.

### 4.2. Portable wearable systems for gait assessment

**Figure 3:**
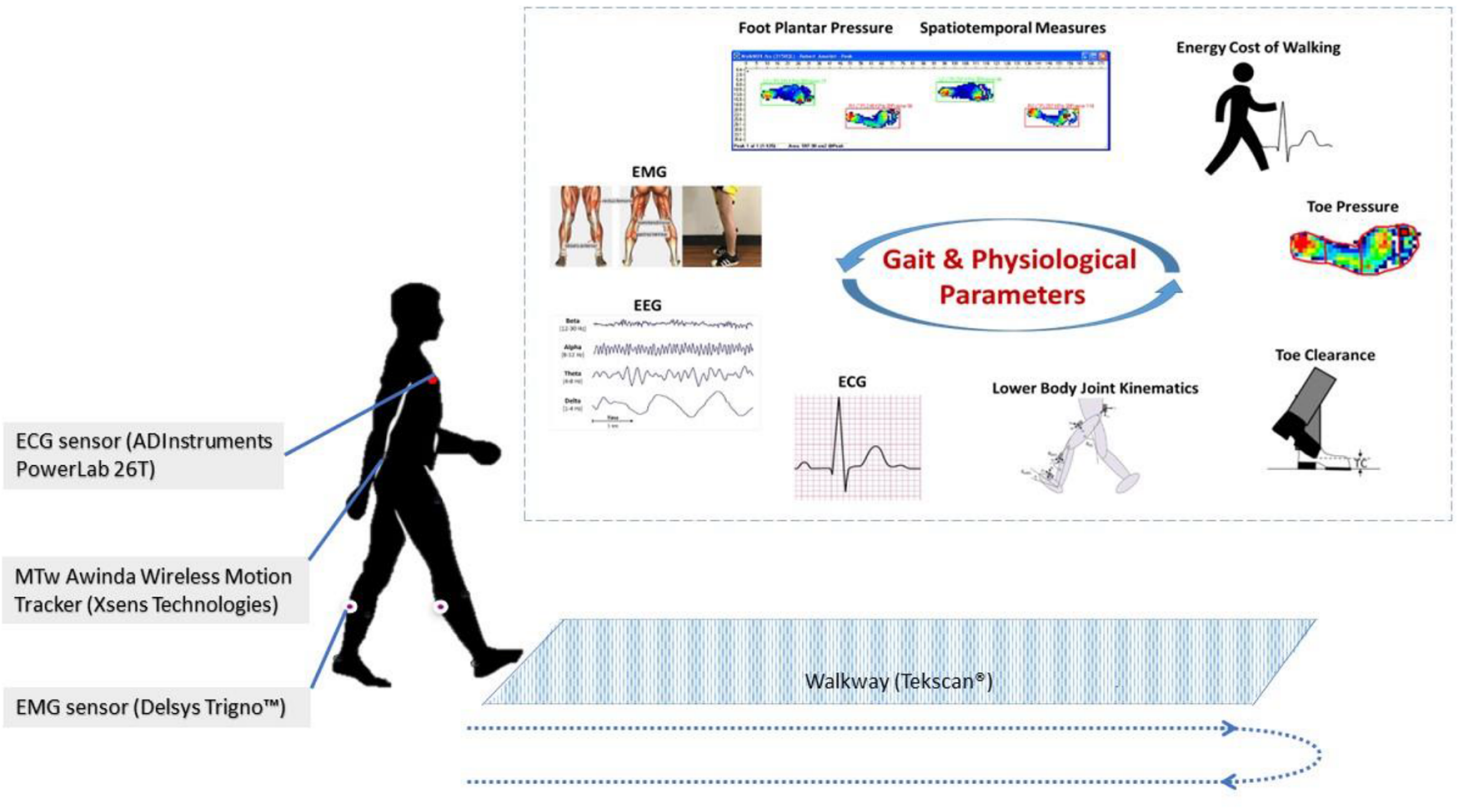
Wearable gait lab

Wearable technology – the use of body-worn sensors to measure the characteristics of human locomotion, has recently emerged as an efficient, convenient, and most importantly, inexpensive option to quantitative gait analysis for both clinical and research-based applications (Figure 3). In general, it uses individual sensor elements, such as accelerometers, gyroscopes, magneto resistive sensors, force/pressure sensors, goniometers, inclinometers, and electromyographic (EMG) sensors, or combined as an inertial measurement unit (IMU) (66). In comparison to conventional counterparts (e.g., walkway and camera based Mocap), wearable sensing enables continuous gait monitoring (> 2 h) outside the lab or clinic, allowing for replication of natural patterns of walking. Moreover, gait patterns over an ample distance could be measured as opposed to limited walking distance in a lab-based setting.

Accelerometers are often used in gait analysis for assessing stability and risk of fall. In a study which used a single tri-axial accelerometer mounted on the sacrum to analyze the risk of fall among 80 participants, accelerometry-based techniques were found to be able to detect subjects with increased risk of fall by employing appropriate machine learning techniques (66). In (67), a 3D accelerometer attached to the lower back was used for stability assessment of older adults. The applicability of a single accelerometer, worn on the back was further examined in (68), which highlights promising results for implementation in routine clinical practices. Considerable work has also been carried out to assess the consistency of gait characteristics obtained from accelerometers, where discrepancies in sensors positioning yield to critical errors (69). Furthermore, in (70), the authors have provided a comprehensive review on the use of accelerometry-based gait analysis techniques and their application to clinical settings.

Gyroscopes are also increasingly employed for gait studies. These devices measure angular velocity and are often combined with accelerometers and other micro-electromechanical systems (MEMS) devices to enhance performance through sensor fusion techniques. They have found applications in step detection, gait event detection, segmental kinematics, and more. For instance, a single gyroscope placed in the instep of the foot was successfully used to detect gait events, including heel strike, foot flat, heel off, and toe-off (71). Another study involved two gyroscopes, mounted on the lower left and right side of the waist to calculate walking steps and step length (72).

Magnetometers measure the magnetic field direction and intensity at a specific point. In combination with other inertial sensors (accelerometers and gyroscopes), they form a so-called inertial measurement unit (IMU), which can produce a drift-free estimation of gait parameters (73). Sophisticated commercialized IMUs (Physiolog 5 IMU, Gait Up, Switzerland, MTw Awinda, Xsens Technologies B.V., Netherlands), as well as in-house developed systems, were equally used for gait studies (74). In the context of human motion analysis, IMUs are employed for several possible goals, for example, to estimate the joint angles (74), to detect the risk of fall in an elderly population, long term monitoring of activities and symptoms (75), measurement of gait events, spatiotemporal parameters (76, 77, 78), ground reaction forces and moments (79), and estimation of gait symmetry (80). Mariani et al. (2010) used IMUs to measure foot kinematics in a study involving both young and elderly and reported the suitability of the system to clinical practice (81). Parisi et al. developed a low-cost system with a single IMU attached to the lower trunk to examine the gait characteristics of both hemiparetic and normal control subjects through measurement of spatiotemporal parameters, which showed excellent correlation with the parameters obtained from a standard reference system (78).

Insole systems for gait measurement and analysis represent a major category, which is cost-effective, portable, and applicable for both indoor and outdoor settings. Over the years, various technologies were developed (82), tested, and commercialized. These include capacitive sensors (Pedar system, Novel GmbH, Germany) (83), force-sensing resistors (FSR) (F-Scan, Tekscan Inc., United States) (84), and piezoresistive sensors (FlexiForce system, Tekscan, United States and ParoTec system, Paromed, Germany) (82). Researchers have adopted different approaches about the design, fabrication, and applications of insole systems. Both prefabricated and in-house fabricated insole systems have been tested for healthy and pathological gait (85, 86). Some studies have also integrated inertial measurement units (IMU) with shoe insoles to enhance their capabilities. Despite the fact that these shoe-based systems have successfully been used for various gait analysis applications, they suffer from some drawbacks, such as (i) distortion of the flexible contact surface due to repeated loading, which leads to changes in the sensor response, (ii) drift in the output due to prolonged load application that causes heat inside the shoe, and (iii) need for subject-specific calibration that may alter accuracy (87). Mancinelli et al. (2012) presented ActiveGait – a novel sensorized shoe system for real-time monitoring of gait deviations associated with Cerebral Palsy in children. They reported that the severity of gait deviations can be estimated with an accuracy greater than 80% using the features derived from the center of pressure trajectories gathered from the shoe system (88). In (87), the authors designed a novel flexible foot insole system using an optoelectronic sensing technology for monitoring plantar pressure deviations in real-time. The system consists of an array of 64 sensing elements and onboard electronics for signal processing and transmission. Experimental validation was conducted on healthy subjects while walking at self-selected slow and normal speed. A commercial force plate (AMTI, Watertown, United States) was used as a reference system for benchmarking. Jagos et al. (2017), on the other hand, developed the eSHOE, which consists of four FSR sensors, a three-axis accelerometer, and a three-axis gyroscope, and reported good agreement with the gait parameters obtained from the GAITRite mat (89). Various other studies have also examined the applicability of shoe-based systems for gait analysis (85, 90–92).

Another class of sensors that found major applications in gait studies is electromyography (EMG) sensors. Surface EMG is a non-invasive technique used to measure muscle activity. In (93), Lee et al. proposed a method using EMG signals to obtain biometrics from gait for personal identification methods. Another study adopted EMG techniques to understand the co-contraction patterns of thigh muscle during free walking using surface EMG (94). These research efforts emphasize the importance of wearable sensors in the study of human gait. The wearable systems discussed in this section are summarized in Table 6.

**Table 6:** Instrumented gait analysis (IGA) systems and their features.

	Instrumented walkway	Camera-based motion capture system	Shoe insole	Wearable technology
Hardware	Low-profile floor walkway with cabling to connect to the computer for data acquisition.	A group of cameras with cabling connected to a data acquisition box and then to a computer. Usually coupled with force plate for gait analysis.	Sensor pads or insoles that cover the entire plantar surface or certain areas of the foot. It can be tethered to a PC or connected *via* Bluetooth or operate with SD cards.	Multiple portable lightweight sensors attached to body segments, charging or receiving station or dongle, and computer.
System setup	The mat can be placed on the floor. Calibration of the system is required prior to any trials.	Cameras can be fixed onto a wall or on tripods. Precise positioning of the cameras and dynamic calibration are pre-requisite. If force plates are used, they should be placed within the capture volume.	Calibration of the system is required before any trials.	Individual sensors should be docked in the station for charging. Ensure that sensors are wirelessly connected, synchronized, and ready to measure/store/transmit data.
Subject setup	No subject setup is required.	Reflective markers are attached to body segments and a calibration trial is performed.	Subjects are required to wear the insole/shoe and perform calibration.	Sensors are placed onto body segments, depending upon the type of manufacturers, an initial setup might be required. Most sensors are factory calibrated.
Advantages	Less setup and data analysis time, easy to operate, require minimal training.	High performance and accurate data, facilitate gait analysis of complex trials involving motion in multiple planes.	Low cost, portable, applicable for indoor and outdoor trials, no dedicated space required for data collection.	Less setup time, low cost, lightweight, and portable. Applicable for indoor and outdoor trials, no dedicated space is required for data collection.
Disadvantages	Non-portable, high cost, require specific data collection space, restricted to over-ground gait analysis, limited to a fixed distance.	Non-portable, high cost, high setup, and calibration time, require dedicated indoor space for data collection, require trained operators.	Low accuracy compared to pressure platforms.	Require specialized algorithms for data processing, which is key to performance and data accuracy.
Examples	Walkway (Tekscan Inc., United States)	Vicon (Vicon Motion Systems Ltd, United Kingdom), Qualisys Pro Reflex system (Qualisys AB, Sweden)	F-Scan (Tekscan Inc., United States)	Physiolog 5 IMU (Gait Up, Switzerland), MTw Awinda (Xsens Technologies B.V., Netherlands)

Although emerging new wearable technologies promise to enhance gait assessment and rehabilitation, there is limited research on the use of wearable technology to assess gait and mobility and its efficacy in clinical settings. According to a recently published review by Peters et. al. on the use of wearable technology to assess gait and mobility in stroke patients (95), most of the available studies are intervention studies conducted in laboratory settings that have used sensors to investigate change in cadence, step time variability, and gait speed. As wearable technologies continue to progress in affordability and accessibility, it is expected that such technologies would enable the gathering of movement-related data in “real-world” and various clinical settings. Importantly, these researchers indicated that so far only a limited number of studies examined reliability and validity of existing wearable devices, highlighting the need for more studies to examine psychometric and other properties when collecting gait and mobility information to determine which wearable technologies are most effective. Another recent review on the evaluation of the use of wearables in PD also indicates that novel technologies and wearables have the potential to enable early or differential diagnosis of PD, monitoring of motion state, prevention, or reduction of off-stage status, and assessing of movement complications. On the other hand, more research is required for the validation and the identification of more accurate markers of PD progression (96). Importantly, these authors warn that wearable devices may not be appropriate in cases of severe motor impairment, off-stage state, cognitive impairment, and for elderly patients and that further research is required for clinical validation.

### 4.3. Wearable-based gait computational algorithms

Besides sensor technology, sensor fusion algorithms play a critical role in predicting the accuracy/precision of these wearable-based systems. Most of the research has focused mainly on gait feature detection, daily physical activity monitoring, and gait data classification targeting disease diagnosis and user recognition. These algorithms are based on different data mining and AI technology, including machine learning, fuzzy computing, wavelet transforms, genetic algorithms, and data fusions. Alaqtash et al. (97) developed an intelligent fuzzy computational algorithm for characterizing gait in healthy, as well as impaired subjects. McCamley et al. established a method to calculate initial and final contact of gait using continuous wavelet transforms, employing waist-mounted inertial sensors (98). Another study cited the use of a single accelerometer mounted at the lower trunk and a corresponding algorithm to identify gait spatiotemporal parameters (68). A real-time gait event detection algorithm was proposed in (99) making use of adaptive decision rules. Further in (100), an original signal processing algorithm is developed to extract heel strike, toe strike, heel-off, and toe-off from an accelerometer positioned on the feet.

A novel gyroscope only (GO) algorithm was proposed in (101) to calculate knee angle through the integration of gyroscope-derived knee angular velocity. A zero-angle update algorithm was implemented to eliminate drift in the integral value. In addition, published work on noise-zero crossing (NZC) gait phase algorithm was also adapted. This method is applicable for continuous monitoring of gait data. Nukala et al. used support vector machines (SVM), KNN, binary decision trees (BDT), and backpropagation artificial neural network (BP-ANN) to classify the gait of patients from normal subjects, where features extracted from raw signals from gyroscopes and accelerometers were used as inputs. This study reported the highest overall classification accuracy of 100% with BP-ANN, 98% with SVM, 96% with KNN, and 94% with BDT (102).

Li et al. proposed DTW algorithm, sample entropy method, and empirical mode decomposition to calculate 3 main gait features of post-stroke subjects: symmetry, complexity character, and stepping stability. A k-nearest neighbor (KNN) classifier trained on the acquired features showed a promising result (area under the curve (AUC) of 0.94), which suggests the feasibility of such techniques to automatic gait analysis systems (43). Rastegari et al. employed a feature selection technique called maximum information gain minimum correlation (MIGMC) to extract gait data of subjects with Parkinson's Disease (103). The performance of several machine learning classifiers, including Support Vector Machines, Random Forest, AdaBoost, Bagging, and Naïve Bayes were also assessed to test the power of the feature set obtained.

The use of novel computational platforms, including Machine Learning, Support Vector Machine, and Neural Network approaches, are increasingly commanding greater attention in gait and rehabilitation research. Although their use in clinical settings are not yet well leveraged, these tools promise a paradigm shift in stroke gait quantification and rehabilitation, as they provide means for acquiring, storing and analyzing multifactorial complex gait data, while capturing its non-linear dynamic variability and offering the invaluable benefits of predictive analytics (1). A recent review article discussed the potential value of ML in gait analysis towards quantification and rehabilitation (104). The authors concluded that further evidence is required although preliminary data demonstrates that the control strategies for gait rehabilitation benefit from reinforcement learning and (deep) neural-networks due to their ability to capture participants' variability. This review paper demonstrated the success of ML techniques in detecting gait disorders, predicting rehabilitation length, and control of rehabilitation devices. Further work is needed for verification in clinical settings.

### 4.4. Data-driven gait rehabilitation in clinical settings

Quantitative gait assessment is invaluable towards disease-specific and patient specific rehabilitation/therapeutic interventions. Spatiotemporal, kinematic, and kinetic parameters obtained during instrumented gait assessment can help clinicians benchmark, devise strategies, and evaluate the effect of various rehabilitation interventions. Gait disorders not only affect these parameters, and patterns and time spent in the various gait phases, but can also highly impact gait symmetry, and regularity, depending on the disease and severity (105). Increasing evidence supports a data-driven physical rehabilitation approach to the treatment of functional gait disturbance (106). There are multiple examples in literature on the effective use of quantitative gait measures towards more effective data-driven rehabilitation. A recent review by Biase et al. (107) studied the most relevant technologies used to evaluate gait features and the associated algorithms that have shown promise to aid diagnosis and symptom monitoring towards rehabilitation in Parkinson's disease (PD) patients. They reported physical kinematic features of pitch, roll and yaw rotations of the foot during walking, based on which feature extraction and classification techniques, such as principal component analysis (PCA) and support vector machines (SVM) method were used to classify the PD patients. They also used gait features, including step duration, rise and fall gradients of the swing phase, as well as standard deviation of the minima as quantitative measures, for benchmarking and monitoring PD motor status during rehabilitation. Interestingly, this review sheds light on need to change the evaluated gait features as a function of disease progression. Another study was Pistacchi et al. (108) suggested spatiotemporal gait parameters, such as speed and step length, where reduced step length seems to be a specific feature of Parkinson's disease gait particularly in early disease stages. On the other hand, asymmetry, step shuffling, double-limb support and increased cadence are more common in mild to moderate stages, while advanced stages are more frequent freezing of gait (FOG) and motor blocks, reduced balance and postural control, motor fluctuations and dyskinesia (109). Researchers have also investigated the evaluation of ambulatory systems for gait analysis post hip replacement (110). They found gait characteristics such as stride length and velocity, as well as thigh and shank rotations different from healthy individuals and recommended their use to monitor post-surgical rehabilitation efficacy. Spatiotemporal gait parameters, such as step length, width and cadence have been used (111) to assess the effect of swing resistance and assistance rehabilitation on gait symmetry in hemiplegic patients. Investigators have also studied whether specific variables measured routinely at a rehabilitation center were predictors of gait performance of hemiparetic stroke patients (112). They found that motor control and balance were the best predictors of gait performance. A recent review article on assessment methods of post stroke gait suggests that multiple spatiotemporal, kinematic, and kinetic parameters can be useful in diagnosing post-stroke gait dysfunction and as quantitative measures to evaluate rehabilitation outcomes (1). Spatiotemporal characteristics of post-stroke gait include reduced step or stride length, increased step length on the hemiparetic side, wider base of support, greater toe-out angle, reduced walking speed and cadence. Stride time, stance period on both lower limb, and double support time are also increased, in addition to less time in stance and more time in swing phase for the paretic side, as well as asymmetries in spatial and temporal factors. Kinematic parameters associated with hemiplegic gait (reduced mean peak extension of the hip joint in late stance, alterations in the lateral displacement of the pelvis and flexion of the knee, and decreased plantarflexion of the ankle at toe-off, in addition to a significant decrease in peak hip and knee flexion during the swing phase, reduced knee extension prior to initial contact, as well as decreased ankle dorsiflexion during swing), and kinetic parameters (asymmetric patterns, as well as decreased amplitudes of the joint moments and joint powers at the hip, knee, and ankle joints on the paretic side) can be used as quantitative means to design and evaluate effective rehabilitation (113–115). IGA has also been successfully used to quantify and improve gait dysfunction associated with ageing and assess the risk of falling (116). Spatiotemporal gait parameters such as velocity, swing time, stride length, stride time- and double support time variability, as well as heel strike and toe off angles, and foot clearance, have been suggested as plausible indicative quantitative measures (116) to assess the risk of falling in elderly subjects. Inertial sensor-equipped shoes additionally provided heel strike and toe off angles, and foot clearance (116). The study (117) summarizes that multi-component exercise therapy which consisted of strength, ROM exercise, balance, flexibility and stretching exercises, circuit exercise training, and gait training was found to enhance gait function for individuals suffering with diabetic peripheral neuropathy compared to control groups using spatiotemporal gait parameters like velocity, cadence, step length, step time, double support time, stride length, stride time, ankle ROM. Gait assessment has potential to develop patient training paradigms for overcoming gait disorders (111).

## 5. Mobile gait lab for clinical applications and beyond

In recent decades, the healthcare field has witnessed a tremendous interest in the use of wearable sensing modalities and AI-driven data management/analysis techniques for patient diagnosis, monitoring, and rehabilitation. The portability, lightweight, ease of use, and high-power efficiency are some of the factors that promote applicability to a clinical platform.

There are few examples in literature demonstrating the potential success of using wearable-based systems for gait assessment in clinical settings. Prajapati et al. assessed the walking activity of inpatients with subacute stroke using commercial accelerometers attached above the ankle. They found that the walking bouts were shorter in duration and gait was more asymmetric (118). Studies have established test-retest reliability and accuracy of different sensor technologies; however, further validation trials are recommended prior to any clinical use. Hsu et al. assessed the test-retest reliability of an accelerometer-based system with infrared assist for measuring spatiotemporal parameters, including walking speed, step length, and cadence, as well as trunk control parameters, including gait symmetry, gait regularity, acceleration root mean square, and acceleration root mean square ratio of healthy subjects in hospital (119). This study showed excellent test-retest reliability of the parameters considered, and thus highlighting the reliability of an infrared assisted, trunk accelerometer-based device for clinical gait analysis. Another study investigated the concurrent validity and test-retest reliability of gait parameters (cadence, gait velocity, step time, step length, step time variability, and step time asymmetry) acquired from elderly subjects, using a tri-axial accelerometer attached to the center of body mass (120). In comparison to a reference GAITRite system, the acquired parameters showed good validity and reliability. Poitras et al. performed a systematic review of 42 studies assessing the reliability and validity of wearable sensors, specifically, IMUs, for quantifying the joint motion (121). Evidence suggests that IMU could be an alternative solution to an expensive motion capture system, as it shows good validity for lower-limb analysis involving fewer complex tasks. However, more work is needed to draw a better conclusion with regards to its reliability, as well as to standardize the protocol to get more accurate data in a clinical setting. Importantly, additional research efforts are needed to examine the responsiveness of wearables in free-living conditions in hospital settings.

## 6. Limitations

This review aimed to summarize available published work on the present and future of gait analysis in clinical settings. The focus was to highlight current systems, scales, and indices, as well as recent technology-driven gait characterization and analysis approaches and their applicability to clinical settings. Within this context, pathological gait associated with different disease, as well as ageing was briefly discussed. As such, this article may have not covered the complete spectrum of gait pathologies and associated parameters. A scoping (non-systematic) search methodology was selected to broaden the scope and integration of the three main aspects of focus (gait pathology, clinical assessment, recent tools, and technologies). In addition, we do not recommend any specific protocol over the other, as most of the papers incorporate different inclusion/exclusion criteria for subject selection, as well as different sampling sizes, which may render comparisons unrealistic.

## 7. Conclusive remarks and future work

This scoping review aimed to shed light on the status of gait assessment in clinical settings, as well as the state-of-the-art emerging tools and technologies and their potential clinical applicability. Clinical gait analysis continues to rely mainly on observational gait and quantitative scales and is hence subjective and suffers from variability and the lack of sensitivity influenced by the observer's background and experience. Based on the reviewed literature, quantitative IGA-based gait analysis, commonly used in research labs, has the capability of providing clinicians with accurate and reliable gait data for informed diagnosis and continuous monitoring. On the other hand, several factors, including high cost and infrastructure challenges; data variability, complexity, and multidimensionality; lack of sufficient knowledge and standardized training in clinical environments; and time constraints, continue to limit its wide-spread deployment. Rapidly emerging smart wearable technology and AI, including Machine Learning, Support Vector Machine, and Neural Network approaches, are increasingly playing a bigger role in gait assessment. Although their use in clinical settings is not yet well leveraged, these tools promise an unprecedented paradigm shift in the quantification of gait in the clinic and beyond, as they provide means for acquiring, storing, and analyzing multifactorial complex gait data, while capturing its non-linear dynamic variability and offering the invaluable benefits of predictive analytics.

Researchers are also paying increased attention to multisource and multi-modality sensor fusion approaches, which can further add value by integrating the output of multiple sensors to capture the complexity and variability of gait. Multimodality sensor fusion also allows for simultaneous monitoring of various physiological signals during locomotion, such as EMG, ECG, and EEG, where fusing these with various gait measures (spatiotemporal, kinematic, and kinetic) can shed light on underlying health conditions and disease etiology towards better informed outcome prediction and clinical decisions. As the volume of data from the variety of sensors, including electroencephalography, electro-oculography, electro-cardiography, and electromyography, motion capture and force sensors data, substantially increases, more AI-driven sophisticated data management and modeling are needed to quantify and interpret complex network AI/NN models. Models which include static and dynamic features, combined with sophisticated data reduction and individualized feature selection of the most relevant gait characteristics are needed to close the loop for this paradigm shift. Future work is warranted on a multidisciplinary level: to validate the clinical applicability and integration of the various sensing modalities, to ensure proper synchronization of the various systems for accurate continuous real-time monitoring, to develop and validate fast and reliable computational platforms, and to implement modular user-friendly interfaces easy to use in any environment.

In summary, instrumented gait analysis is a well-established tool for the quantitative assessment of gait dysfunction which could be effectively used for functional diagnosis, treatment/surgery/rehabilitation/planning, and progression monitoring for a wide spectrum of disease. The literature indicates that recent advancement in wearable technology and computationally advanced data analytics, including AI, can overcome the challenges of traditional gait labs, allowing for less costly, portable, and relatively simple gait testing protocols in clinical settings, as well as user-friendly data management, analysis, and interpretation computational platforms. On the other hand, the development of clinically driven standardized methodology and procedures is of paramount significance and remains largely unaddressed. These standardized practices should not only focus on quantitative gait diagnosis but should also incorporate sophisticated objective measures and 3-D dynamic gait profiles and markers for monitoring progress and outcome prediction and evaluation. Proper gait protocols should be devised and leveraged towards identifying gait characteristics that could be effectively used as early disease diagnostic markers. Importantly, training clinical teams at various levels, from doctors and surgeons to physiotherapists and other allied health professionals, on properly using these novel assessment and computational tools is equally important and warrants an equally rapid paradigm shift in training and practice in clinical settings towards patient-specific precise medicine.
